# Review of “Contagious Venereal Disease Amongst Horses in Kent County, Canada”

**Published:** 1890-05

**Authors:** W. L. Williams

**Affiliations:** V.S. (Montreal); Bloomington, Ill.


THE JOURNAL
OF
COMPARATIVE MEDICINE AND
VETERINARY ARCHIVES.

VOL. XI. MAY, 1890. No. 5.

### REVIEW OF "CONTAGIOUS VENEREAL DISEASE AMONGST HORSES."

BY W. L. WILLIAMS, V.S. (MONTREAL).
BLOOMINGTON, ILL.

INTEREST is once more revived in the subject of the venereal diseases of horses by the recent official publication by the Ontario government of a monograph entitled, "Report on the Outbreak of Contagious Venereal Disease Amongst Horses, in the County of Kent, Ontario, by Peter H. Bryce, M.A., M.D., Commissioner, Toronto, 1889," which has been inserted in the JOURNAL OF COMPARATIVE MEDICINE AND VETERINARY ARCHIVES, for March and April, 1890.

This subject was first brought prominently before the English-speaking veterinary world in 1887, by the announcement that the most dreaded of these maladies, equine syphilis, existed in Illinois, this being the first authentic outbreak reported in an English-speaking country, although we had been forewarned somewhat of its character through the writings of Fleming and others.

This report must necessarily attract its share of attention, as Dr. Bryce occupies the honorable position of secretary of the Provincial Board of Health of Ontario, and was selected over the heads of veterinarians by the Ontario Government, under Royal Commission, to investigate a somewhat extensive outbreak of disease among animals in a province where there are probably more veterinarians to its area than in any other province or state in America, and having in its capital (the home of Dr. Bryce and Minister of Agriculture Drury, to whom the report is addressed), a veterinary college which annually sends from its halls more veterinarians than any other school in Christendom ; and which is presided over by an old and honored graduate of one of the leading veterinary colleges of the English-speaking world ; and with a number of veterinarians in the immediate territory affected, who had had opportunities for studying venereal diseases of horses for several years.

His selection for the investigation of this important epizootic would naturally suggest an unwritten special fitness not indicated by his M.A. and M.D. degrees, and will heighten the interest in his report, and it invites a critical study.

On his initial page he sounds the keynote of the whole by embracing that antiquated theory of the medical profession, that all medical science belongs to and emanates from human medicine ; a theory which, thanks to the rapid advancement of our own profession, is now cherished by a very small and ever decreasing minority of the leading doctors of medicine ; and after declaring all veterinary literature upon the subject of venereal diseases of horses worthless, he finds it necessary to revert to human medicine, in which "extended studies" of these have been made, that he may "set forth in some systematic manner the principal biological and pathological phenomena of venereal diseases in horses so far as they have appeared in this county."

He casts aside, without remark, the experiments of Houzaud, Peuch, Trasbot, Blaise and others, by which they have shown that the chief venereal diseases of man and horse, although analogous, are yet distinct, and that consequently any decisive study of these maladies must be made upon the patient affected ; nor does he offer any explanation for his failure to make an elaborate biological study of the Kent County venereal disease instead of adopting the ideas of medical writers ; and although the main part of his report is a biological dissertation, he fails to state what, if any, micro-organisms were present in the morbid products.

The author then turns to *one* venereal disease of man—gonorrhœa—and by its literature attempts to elucidate *all* venereal diseases of horses, unfortunately, we think, remembering that gonorrhœa is the most unimportant venereal disease of horses ; and he is still more unfortunate in selecting for his dissertation a disease which in man is essentially non-eruptive, whereas the Kent County outbreak was chiefly characterized by abundant vesicular or papular eruptions.

After abundant quotations from medical authorities, he concludes that human gonorrhœa is due to the invasion of the staphylococcus pyogenes aureus (pus-forming micro-organism), with the possible co-existence of a specific microbe (gonococcus), and further concludes that his quotations afford a reasonably clear explanation of all the phenomena exhibited in the Kent County outbreak, without the necessity of showing that this or any other micro-organism existed therein.

Upon this biological foundation, under the head of "Prophylactic Regulations," he says: "Manifestly, therefore, a period of quarantine similar to that at present exercised in the matter of imported cattle is desirable."

This is certainly a bold step in advance of recognized sanitary measures, when he must admit that the staphylococcus which he claims causes the venereal diseases of horses is as widely diffused as the animal kingdom, existing not alone in the discharges from a gonorrhœic horse or man, but in pus, wherever it may exist, and his reasons do not appear for selecting as a subject for quarantine restrictions those animals only which are suffering from purulent discharges from the genital organs. Nor do we see of what avail his quarantine restrictions can be when he admits that the disease can be generated through sexual contact of animals when one of them is affected with chronic vaginitis, acid vaginal discharges, etc.

Again, Dr. Bryce calls into question a statement made in my report (Report of the State Board of Live Stock Commissioners of Illinois on "Equine Syphilis," 1887), to the effect that eruptions of the genital organs are rarely present in equine syphilis, and in no case of a pathognomonic character, and quotes from my report the description of the "Moore" horse, the *supposed* bearer of disease in the Illinois outbreak, suggesting that I had "forgotten" previous statements regarding destructive changes which I had described as having taken place in the genitals of that animal. But he should have observed that my report does not state that these changes were caused by equine syphilis, and I may add here that I did not at that time believe such to be the case, and have found no reason since for changing my views, so that the quotation from my report is wholly misapplied.

He then misquotes me by pretending to copy from me the following words, which are not found in my report: "I am obliged to take exception to the generally accepted ideas of English writers and translators, and separate the so-called benign form from one of the diseases under consideration as a wholly distinct venereal disorder," and then remarks that "nothing further than these [mis] quotations are needed to show how slight are the grounds upon which Mr. Williams divides the outbreaks which he has investigated into distinct diseases."

Dr. Bryce either failed to consult, or has wholly disregarded, the recent writings of Thanhoffer, Zundel, Blaise, Dieckerhoff, Trasbot and others, in claiming that no adequate grounds for differential diagnosis of the venereal diseases of horses exists in veterinary literature, when all with one accord say that there is no reason for confounding equine syphilis, a malignant fatal disease, with the benign eruptive affection described by me as chancroid, by Fleming as exanthematous disease of the genital organs, by French writers as coïtal exanthem or genital horsepox, by Dieckerhoff and others as phlyctenausschlag, and by Dr. Bryce as a nameless malady due to the ravages of the staphylococcus pyogenes aureus.

As to the question of eruptions on the genitals in equine syphilis and the grounds for differentiation between it and other venereal diseases of horses, the following may be of interest. M. Laquerrie, speaking of a report on venereal diseases in horses by M. Blaise, of the Blidah Remount Depot, Algeria, after detailing the symptoms in twelve affected stallions under close observation, says: "There was a total absence of chancres or cicatrices, symptoms which are very prevalent in syphilis of the human subject, but which although recognized as having an existence in the horse and mare, are still of very infrequent occurrence." *Veterinary Journal*, Vol. XXX, p. 101.

Dieckerhoff (*Spec. Path and Therap.*, Vol. I, pp. 393–405) says: "It is only since the middle of the present century that equine chancroid (bläschenausschlag der geschlechtstheile) and equine syphilis (beschälkrankheit) have been clearly differentiated. . . . The oft-described formation of vesicles and ulcers on the penis, sheath and scrotum occur (in equine syphilis) only rarely. In the vagina of affected mares Rodloff found no vesicles or ulcers. Neither has Maresch personally seen vesicles, but has noted papillomata and excoriations. He adds, however, that these ulcerative patches in mares are of very rare occurrence,'' etc., etc.

Again, the inference Dr. Bryce would have the reader draw from his references to the ''Henry Abraham'' stallion, concerned in the Kent County outbreak, is needlessly erroneous, since he could readily have learned, had he desired, that the horse brought into Canada by Courtney was not one of the full-blooded draft horses registered under that name, but a grade stallion, sired by the imported horse bred by a Mr. Armstrong and sold as a sound horse to Michigan parties, and thence to Mr. Courtney. There being no equine syphilis in the country where he was raised, renders his exposure to that disease impossible, and the other two venereal diseases have so short a career that their importation into Canada by this horse could not be charged to Illinois, when the horse had made several seasons safely after leaving this State ; an intimation rendered more evidently needless by Dr. Bryce's tabulated list of stallions, in which he admits that the Kent County disease had existed in Canada since 1874.

We see why Dr. Bryce should desire to establish such a theory, when he shows that the disease had existed in Canada since 1874, and even had it not existed, Dr. Bryce asserts that the staphylococcus pyogenes is sufficient, under favorable conditions, to establish the disease, and this micro-organism, he will certainly not deny, exists abundantly in Canada.

A careful study of the entire report, including the descriptions of symptoms, course and termination of the cases in Kent County, Ont., as delineated by Dr. Bryce and the veterinarians and others whose evidence is given in the appendix, will necessarily lead the reader to believe that the outbreak of venereal disease in Kent County, Ont., is identical with the affection described by Fleming as exanthematous disease of the genital organs, by the French as coïtal exanthem or genital horsepox, and by myself as equine chancroid. And yet Dr. Bryce tries hard to reconcile the phenomena of the outbreak in question with equine syphilis, dourine or beschälkrankheit.

The Kent County outbreak (like the one at Kempton, Ill., described by me in *An. Ref. State Board Live Stk. Com. of Ill.*,

*1887*) is characterized chiefly by abundant local eruptions, while the Illinois outbreak of equine syphilis, in full accord with all other recent veterinary authorities, was noted for the almost or complete absence of local eruptions. The Kent County outbreak ran a rapid course—two to six or eight weeks—while the Illinois and other outbreaks of equine syphilis extended their course over months and years. The Kent County outbreak, including thirty-three stallions and an indefinite number of mares, ran a benign course, without an authentic fatality, while in Illinois, like in every land, equine syphilis ended fatally in over 50 per cent. of the affected cases; and of the remainder the great majority were left in such condition that they were readily purchased by the State for from ten to twenty-five dollars per head, for slaughter.

While Dr. Bryce has satisfactorily demonstrated that he is unable to differentiate between the three venereal diseases of horses, he has not shown that it follows that there are no veterinarians competent to make a safe diagnosis between them, and the problem as to what micro-organism caused the outbreak of venereal disease in Kent County, or any other outbreak of this or other venereal disease of animals, remains as far from solution as ever.

In his recommendations as to measures for the control and extirpation of veneral diseases among horses, we discover nothing well calculated to annihilate or subdue the staphylococcus pyogenes aureus in Canada, and until he does his efforts to control its ravages in this one direction must prove futile.

On the whole, Dr. Bryce's report is all that could be expected when an M.D., without previous study, is abruptly called to investigate an important outbreak of disease among animals, which, without adding to our knowledge, can only serve to create unwonted alarm and injury to the horse-breeding industry of the affected territory, and possibly renew, in the minds of some younger members of our profession, the perplexity which, up to recent years, has prevailed in regard to the venereal diseases of horses.

